# Total loss of *VHL* gene function impairs neuroendocrine cancer cell fitness due to excessive HIF2α activity

**DOI:** 10.1073/pnas.2410356121

**Published:** 2024-09-25

**Authors:** Muhannad Abu-Remaileh, Nicole S. Persky, Yenarae Lee, David E. Root, William G. Kaelin

**Affiliations:** ^a^Division of Molecular and Cellular Oncology, Department of Medical Oncology, Dana-Farber Cancer Institute, Harvard Medical School, Boston, MA 02215; ^b^Broad Institute of Massachusetts Institute of Technology and Harvard, Cambridge, MA 02142; ^c^HHMI, Chevy Chase, MD 20815

**Keywords:** paraganglioma, pheochromocytoma, neuroblastoma, von Hippel–Lindau, hypoxia

## Abstract

Loss-of-function germline *von Hippel–Lindau* (*VHL*) tumor suppressor mutations cause VHL disease, which predisposes individuals to kidney cancer, hemangioblastomas, and paragangliomas. The risk that a given VHL disease family will manifest some or all these tumor types is profoundly influenced by the *VHL* allele it carries. For example, almost all VHL disease families that develop paraganglioma have missense *VHL* mutations. VHL families with null *VHL* alleles develop kidney cancer and hemangioblastomas without a high risk of paraganglioma. The latter is surprising because the *VHL* gene product, pVHL, suppresses the HIF2 transcription factor and gain-of-function HIF2 mutations are also linked to paraganglioma. Paragangliomas arise from the sympathetic or parasympathetic nervous system. Given the lack of human paraganglioma cell lines, we studied the effects of inactivating *VHL* in neuroblastoma cell lines, which also arise from the sympathetic nervous system. We found that total loss of pVHL function profoundly impairs the fitness of neuroblastoma cell lines in a HIF2-dependent manner both ex vivo and in vivo. This fitness defect can be rescued by pVHL variants linked to paraganglioma, but not by pVHL variants associated with a low risk of paraganglioma. These findings suggest that HIF2 activity above a critical threshold prevents the development of paraganglioma.

## Significance

The molecular explanations for the different tumor type-specific risks caused by different germline *von Hippel–Lindau* (*VHL*) tumor suppressor gene mutations (genotype–phenotype correlations) are not well understood. For example, it is unclear why missense *VHL* mutations predispose to paragangliomas while null VHL mutations do not. This observation is unexpected because the *VHL* gene product, pVHL, suppresses HIF2 and gain-of-function HIF2 mutations can cause paragangliomas. More broadly, it is unclear why *VHL* mutations are only linked to kidney cancers among common cancers. We found that complete loss of pVHL function suppresses neural crest tumor growth in a HIF2-dependent manner, arguing that HIF2 activity beyond a certain threshold suppresses the growth of paragangliomas, and perhaps many other tumor types.

The *von Hippel–Lindau* (*VHL*) tumor suppressor gene encodes the substrate recognition subunit (pVHL) of an E3 ubiquitin ligase that targets the alpha subunit of the heterodimeric HIF (Hypoxia-Inducible Factor) transcription factor for proteasomal degradation when oxygen is plentiful. Under low oxygen (hypoxic) conditions, HIFα is not recognized by pVHL and becomes more stable. It then dimerizes with HIFβ (more commonly called ARNT) and activates genes that normally play roles in adaptation to hypoxia (1). The binding of pVHL to HIFα is oxygen dependent because it requires that HIFα be hydroxylated by one (or both) of two conserved prolyl residues by members of the EglN (also called PHD) 2-oxoglutarate-dependent dioxygenases. The EglNs have low oxygen affinities and thereby act as oxygen sensors. They are also sensitive to changes in cellular redox and metabolism (2). For example, hydroxylation of HIFα by the EglNs is coupled to decarboxylation of 2-oxoglutarate to succinate, and high levels of succinate (or the related chemical fumarate) inhibit EglN activity (3, 4). Humans have 3 *EglN* genes although *EglN1* is the primary regulator of HIF stability in the cells examined to date (2).

Germline inactivating *VHL* mutations predispose to a variety of neoplasms, including clear cell renal cell carcinoma (ccRCC), blood vessel tumors called hemangioblastomas, and neural crest–derived tumors called paragangliomas (5). Tumor development in this setting is linked to loss or silencing of the remaining wild-type *VHL* allele (6). Somatic biallelic *VHL* mutations (or hypermethylation) are also common in sporadic ccRCCs and have been documented in sporadic hemangioblastomas and paragangliomas (7–9).

Humans have three HIFα paralogs: HIF1α, HIF2α, and HIF3α. HIF1α and HIF2α exhibit both shared and paralog-specific target genes and functions (10). HIF3α, which is less extensively studied, is thought to lack the ability to activate transcription (11–13). Notably, HIF2α, rather than the more widely studied and ubiquitously expressed HIFα paralog, HIF1α, drives the development of ccRCC (14, 15). HIF2α likely also plays a pathogenic role in hemangioblastomas because HIF2α, but not HIF1α, is both necessary and sufficient for the development of vascular proliferative lesions caused by genetic ablation of *VHL* in various mouse organs (16, 17). Moreover, hemangioblastomas, like ccRCCs, respond to the HIF2α inhibitor Belzutifan in humans (18). Indeed, Belzutifan was recently approved for ccRCCs and hemangioblastomas arising in VHL disease.

There are no reliable preclinical models available for studying *VHL^−/−^* paragangliomas and interrogating the role of HIF in such tumors. However, loss-of-function *succinate dehydrogenase* (*SDH*) subunit mutations that cause the accumulation of succinate (which interferes with EglN activity), loss-of-function *EglN1* mutations, and gain-of-function *EPAS1* mutations (encoding HIF2α) have been identified in hereditary and sporadic paragangliomas (8). Hence, based on the law of parsimony, it is likely that HIF2α plays a causal role in *VHL^−/−^* paragangliomas. Moreover, Belzutifan is active against paragangliomas caused by gain-of-function HIF2 mutations (19) and against VHL-associated pancreatic neuroendocrine tumors, which share similarities with paragangliomas (18, 20).

The genetics of VHL disease present two intriguing paradoxes. First, despite the ubiquitous expression of *VHL*, *EglN1*, and *HIF1A* (encoding HIF1α), VHL-associated neoplasms are restricted to specific tissues. Conceivably this is due, at least partly, to the protumorigenic effects of HIF2α relative to HIF1α and the more restricted expression of *EPAS1* compared to the ubiquitous expression of *HIF1A*. Second, there are strong genotype–phenotype correlations in VHL disease, with some families having a low risk of paraganglioma (Type 1 VHL Disease) and some families having a high risk of paraganglioma (Type 2 Disease), including families that present as familial paraganglioma without the usual stigmata of VHL Disease (Type 2C) (1). Curiously, the most deleterious *VHL* mutations with respect to HIF regulation, including true null *VHL* alleles, are associated with Type 1 Disease (1). Conversely, almost all the mutations linked to Type 2 VHL disease are hypomorphic in terms of HIF regulation, and some Type 2C mutants appear wild-type with respect to HIF regulation, at least when overexpressed in preclinical models (1). This suggests two nonmutually exclusive possibilities: first, that HIF activity surpassing a certain threshold is incompatible with paraganglioma development, and, second, that certain *VHL* missense mutations, particularly Type 2C mutants, cause paraganglioma through the loss of a HIF-independent function, either alone or in conjunction with HIF deregulation. Notably, pVHL appears to play both HIF-dependent and HIF-independent roles in regulating apoptosis induction in neural crest cells when prosurvival factors become limiting, such as occurs during the natural culling of excess sympathetic neuroblasts during embryological development (1, 21).

## Results and Discussion

The *VHL* gene, despite being a tumor suppressor gene, scores as a “common essential gene” based on the Broad Institute DepMap data derived from 1,078 cell lines subjected to pooled whole genome CRISPR screens and 710 cell lines subjected to pooled whole genome RNAi screens (22). Therefore, loss of *VHL* reduces the fitness of most cancer cells, at least under standard cell culture conditions ex vivo. Kidney cancer lines, many of which already bear *VHL* mutations, are outliers in this regard (*SI Appendix*, Fig. S1*A*). This is consistent with them being permissive for transformation by *VHL* inactivation.

**Fig. 1.**
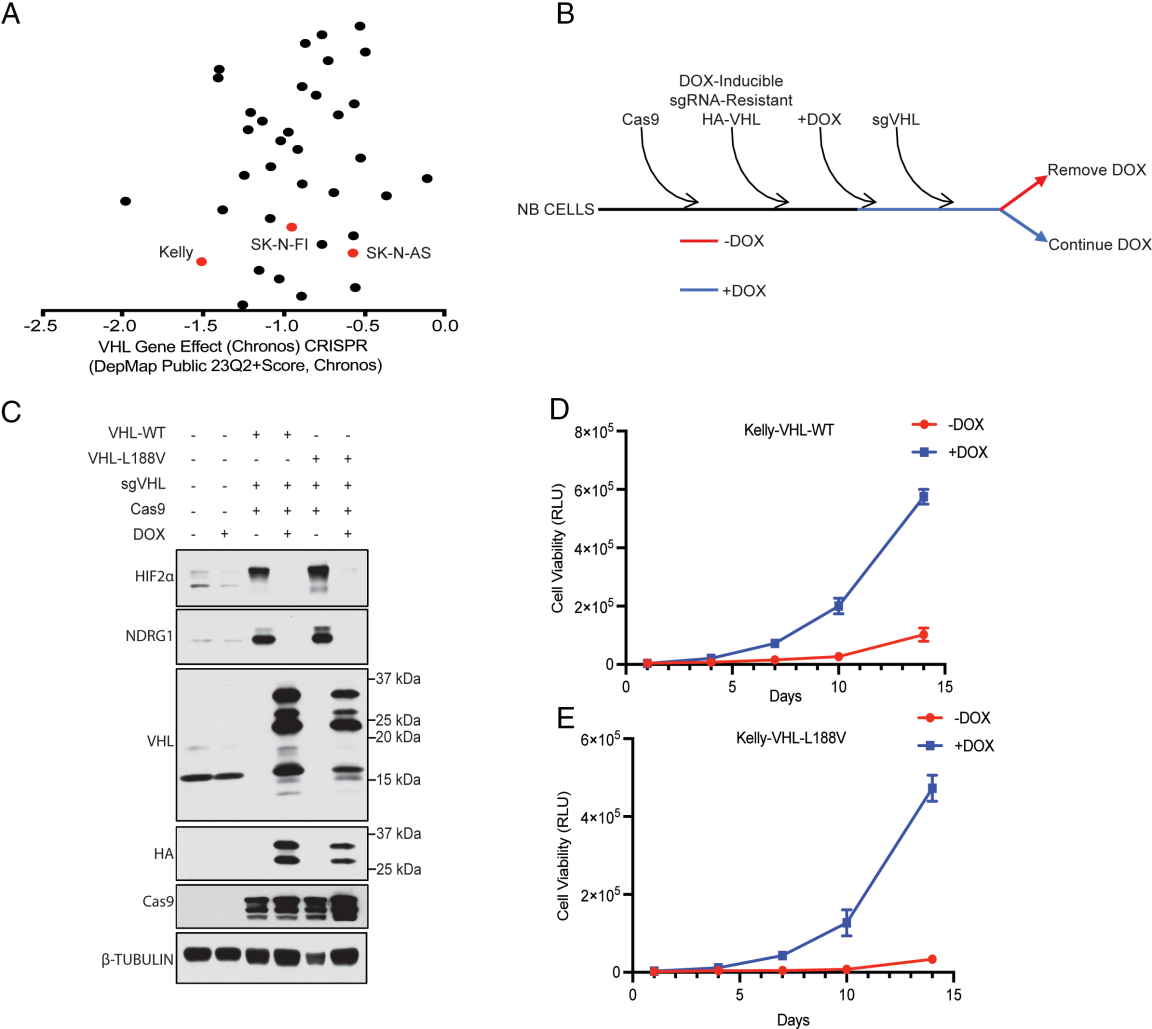
*VHL* inactivation suppresses the proliferation of Kelly neuroblastoma cells. (*A*) Shown are the *VHL* gene effect (chronos) scores, which reflect the dependency on *VHL* based on enrichment or depletion of *VHL* sgRNAs in genome-wide CRISPR screens conducted across 37 neuroblastoma cell lines. The cell lines are arbitrarily distributed along the *y* axis so that each cell line can be seen as a discrete dot. A score of 0 indicates a gene is not essential whereas a score of −1 or less corresponds to essential genes. (*B*) Schematic for sequential stable infection of Kelly neuroblastoma cells to 1) express Cas9, 2) a sgRNA-resistant *VHL* cDNA under the control of a doxycycline (DOX)-inducible promoter, and 3) a *VHL* sgRNA. Immunoblot analysis (*C*) and proliferation curves based on CellTiter-Glo2.0 (*D* and *E*) of Kelly cells expressing DOX-inducible wild-type pVHL or pVHL L188V, generated as in (*B*), that were grown in the presence (“+”) or absence of DOX ("−"). In (*C*) the HIF2-responsive gene product NDRG1 was included as a control. In (*D* and *E*) DOX was or was not withdrawn at time zero. For all panels, data presented are means ± SEM.

There are no paraganglioma cell lines in the DepMap cell line collection, and we are unaware of any faithful human paraganglioma cell lines. Pheochromocytomas are intra-adrenal paragangliomas. In our hands, hPheo1 cells (23), which are purported to be pheochromocytoma cells, do not express the dopamine β-hydroxylase and tyrosine hydroxylase markers expected of paraganglioma cells under our cell culture conditions (*SI Appendix*, Fig. S1*B*). We noticed that multiple neural crest–derived cancer cell lines, including neuroblastoma and melanoma lines, had conspicuously high DepMap *VHL* dependency scores (Fig. 1*A* and *SI Appendix*, Fig. S1*A*). We found that Kelly neuroblastoma cells, in addition to being *VHL*-dependent, expressed both dopamine β-hydroxylase and tyrosine hydroxylase, in contrast to the SK-N-FI and SK-N-AS neuroblastoma cell lines (*SI Appendix*, Fig. S1*B*). We therefore chose to study Kelly cells further.

Human cells can produce both a long (~28 kD) and short (~19 kD) form of pVHL by virtue of two alternative, in-frame, translational initiation codons (24, 25). In most of the biochemical and biological assays performed to date, these two protein isoforms behave similarly (26). We infected Kelly neuroblastoma cells to stably express Cas9 and a doxycycline (DOX)-inducible cDNA encoding both isoforms of wild-type pVHL with an N-terminal hemagglutinin (HA) epitope tag immediately upstream of the first translation initiation codon. Kelly cells expressing Cas9 and various DOX-inducible tumor-derived pVHL mutants were similarly made in parallel. We then tried to biallelically inactivate *VHL* using CRISPR in the presence of DOX (Fig. 1*B*).

We succeeded in eliminating endogenous pVHL in Kelly cells that were engineered to express exogenous wild-type pVHL or Type 2 pVHL mutants such as the L188V Type 2C variant, Y112H Type 2A variant, and V84L Type 2C variant (Fig. 1*C* and *SI Appendix*, Fig. S1*C*) (27, 28). In contrast, we could not inactivate endogenous pVHL in cells that expressed pVHL C162F, which is associated with a low risk of paraganglioma (Type 1) and is grossly defective with respect to HIF suppression (*SI Appendix*, Fig. S1*C*) (29). The exogenous pVHL variants migrated as multiple bands, likely due, at least in part, to alternative translation initiation and posttranslational modifications such as phosphorylation. The former likely explains the absence of the HA-tag in the faster migrating species. Notably, withdrawal of DOX, and hence loss of exogenous pVHL, arrested the growth of the Kelly cells engineered to produce exogenous wild-type pVHL or the Type 2 pVHL variants (Fig. 1 *D* and *E* and *SI Appendix*, Fig. S1 *D* and *E*). This would explain our inability to eliminate endogenous pVHL in the Kelly cells expressing the grossly defective C162F variant. Similar results were obtained with SK-N-FI (*SI Appendix*, Fig. S1 *F* and *G*), which score as pVHL-dependent in DepMap, but not with SK-N-AS (*SI Appendix*, Fig. S1 *H* and *I*), which are far less pVHL-dependent in DepMap (Fig. 1*A*). A *VHL* cDNA that produces exclusively the pVHL p19 isoform, which is the major endogenous pVHL isoform in Kelly cells, behaved similarly to the *VHL* cDNA encoding both wild-type pVHL isoforms (*SI Appendix*, Fig. S1 *K* and *J*). Significantly, the cessation of DOX administration, resulting in the depletion of exogenous pVHL in Kelly cells modified to express either wild-type pVHL or L188V pVHL, resulted in cell cycle arrest (*SI Appendix*, Fig. S2) and elevated apoptotic cell counts (*SI Appendix*, Fig. S3).

**Fig. 2.**
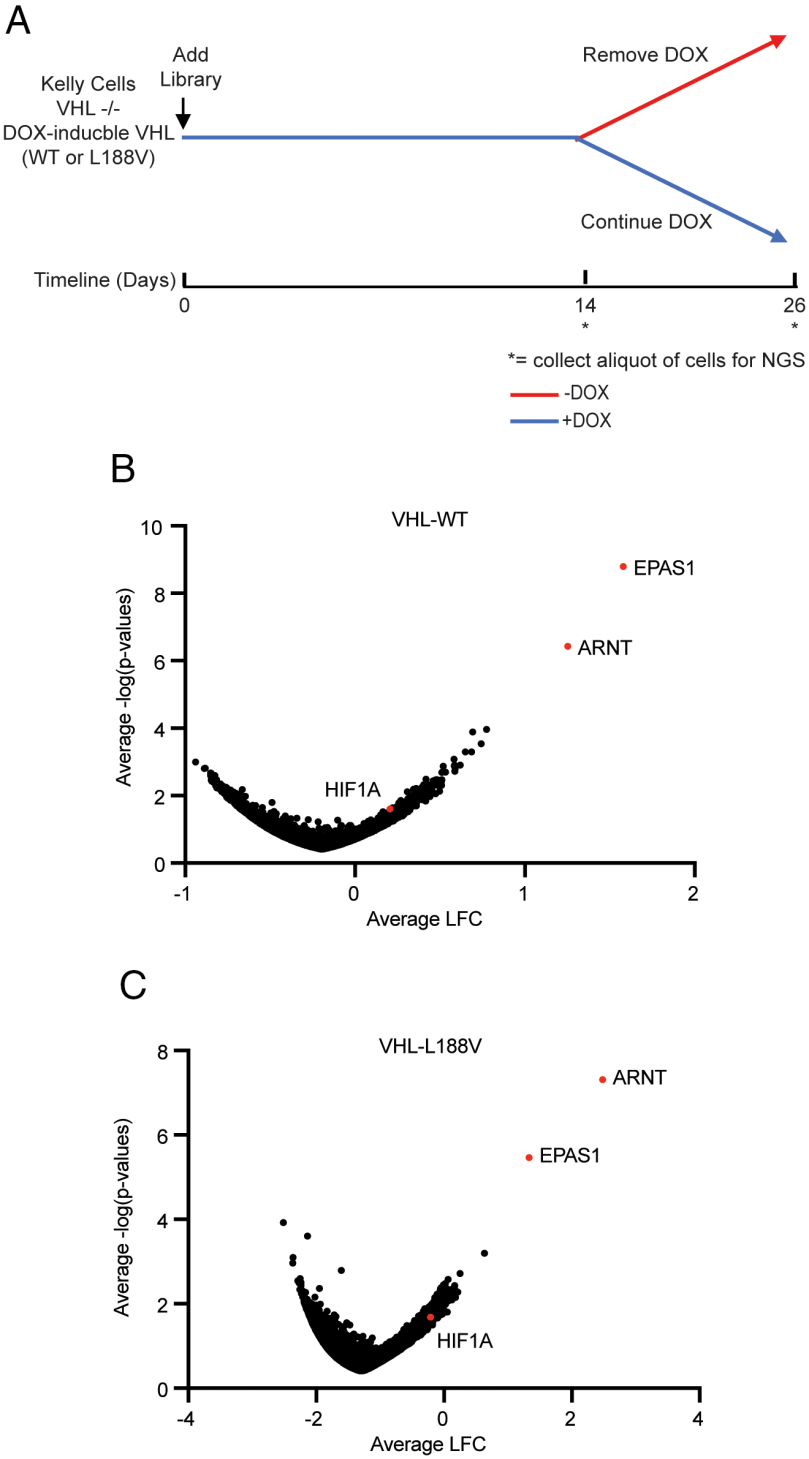
Genome-wide CRISPR screen suggests excessive HIF2α activity impairs the proliferation of Kelly cells lacking pVHL. (*A*) Schema for CRISPR screen. Kelly neuroblastoma cells described in Fig. 1 were infected with the Brunello lentiviral genome-wide sgRNA library with a 0.3 to 0.4 MOI, yielding ~500 cells/sgRNA followed by puromycin selection for 5 d. Nine days later (14 d after infection, referred to as Day 14), an aliquot of cells was harvested for the isolation of genomic DNA (gDNA). The remaining cells were split 50:50 to media that did or did not contain DOX. Twelve days later (26 d after infection, referred to as Day 26), the cells were harvested for gDNA extraction. (*B* and *C*) Volcano plots showing genes whose sgRNAs were enriched or depleted at Day 26 vs. Day 14 after DOX withdrawal in Kelly cells expressing DOX-inducible wild-type pVHL (*B*) or pVHL L188V (*C*).

To begin to understand the mechanism underlying this pVHL dependence, we infected the DOX-inducible wild-type pVHL Kelly cells and DOX-inducible pVHL L188V Kelly cells in 4 technical replicates with the Brunello whole genome CRISPR Knockout (KO) Pooled sgRNA library (30) (19,114 genes, 4 sgRNAs per gene, 500 cells per sgRNA) in the presence of DOX (Fig. 2*A*). Fourteen days after infection (referred to as Day 14), an aliquot of cells was removed and used to isolate genomic DNA. The remaining cells were split 1:1 to media that did or did not contain DOX. Twelve days later (referred to as Day 26), gDNA was again isolated. We then determined sgRNAs abundance by next-generation sequencing of the gDNA samples and looked for genes whose sgRNAs were significantly enriched or depleted in the absence of DOX (“hits”) at Day 26 (endpoint) vs. the distribution at Day 14 (start point).

The top-scoring enrichment hits were *EPAS1* and *ARNT* in both the wild-type pVHL (Dataset S1) and L188V pVHL (Dataset S2) screens (raw data available on Zenodo) (31), although *EPAS1* was clearly the top hit in the former and *ARNT* the top hit in the latter (Fig. 2 *B* and *C*). The significance of this difference in rank order is not clear, but might reflect stable differences in the molecular changes induced by wild-type pVHL vs. L188V pVHL over time prior to the withdrawal of DOX. Interestingly, *HIF1A*, encoding HIF1α, did not score in either cell line, suggesting that the growth suppression observed in Kelly cells after pVHL loss was primarily driven by increased HIF2α activity. Furthermore, in silico analysis of coessentiality with *VHL* across all the cell lines (or across all the cell lines except ccRCCs) in the Project Achilles data of the Broad Institute demonstrated that *VHL* dependence and *EPAS1* dependence are anticorrelated (*SI Appendix*, Fig. S4 *A* and *B*). In contrast, *VHL* dependence correlates with dependence on *TCEB1* (*SI Appendix*, Fig. S4 *A* and *B*), which encodes the pVHL ubiquitin ligase component ELONGIN C, and *EGLN1* (32–34). These findings suggest that HIF2 impairs the fitness of multiple cell types after pVHL inactivation.

**Fig. 3.**
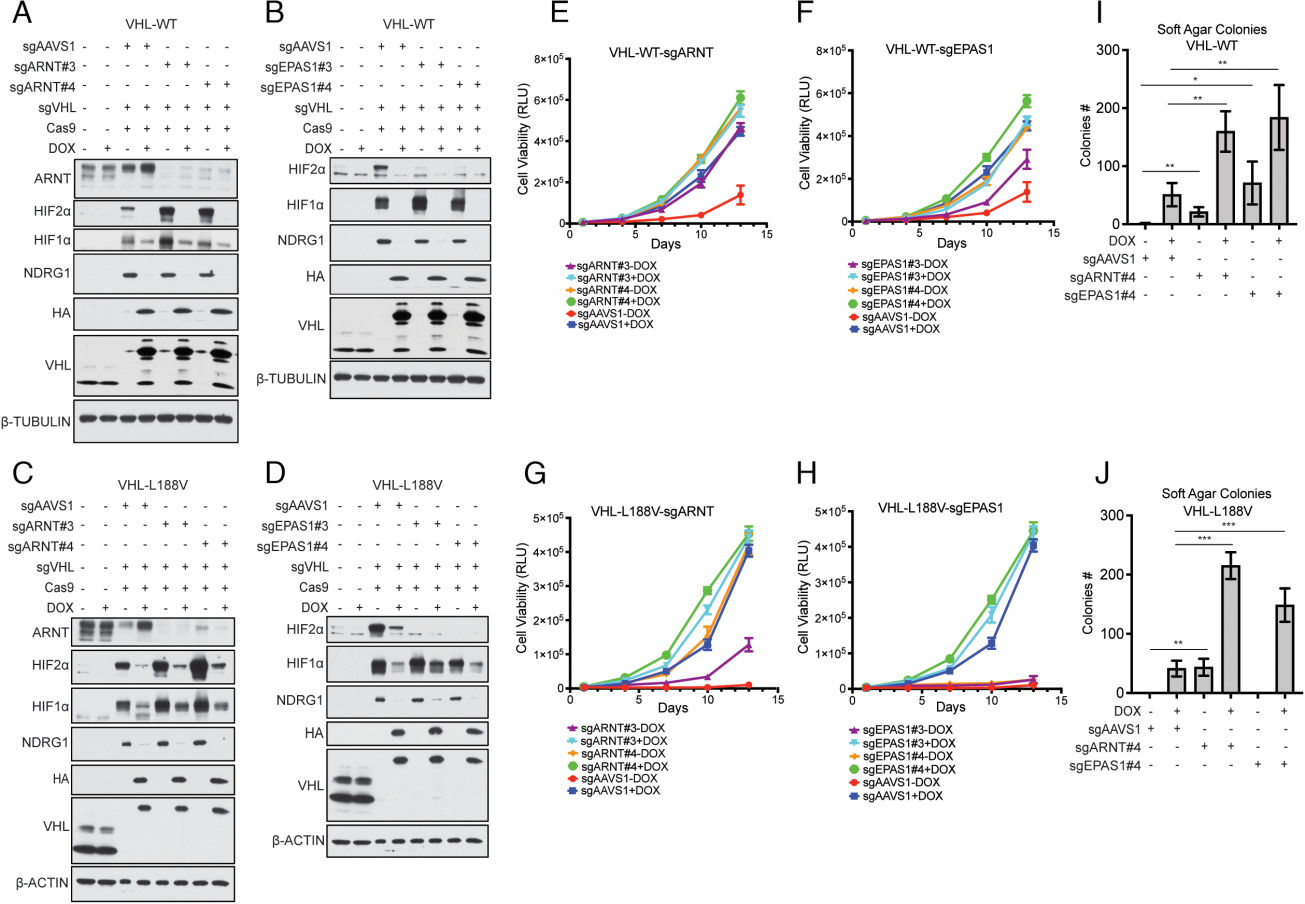
HIF2α is necessary for impaired proliferation in Kelly neuroblastoma cells lacking pVHL. Immunoblot analysis of parental Kelly cell and Kelly cells expressing DOX-inducible wild-type pVHL (*A* and *B*) or pVHL L188V (*C* and *D*) grown in the presence (+) or absence of DOX (−). Where indicated the cells were also infected to express one of two *ARNT* sgRNAs (*A* and *C*), one of two *EPAS1* sgRNAs (encoding HIF2α) (*B* and *D*), or a control AAVS1 sgRNA (*A–D*). (*E* and *F*) Proliferation curves of the cells studied in (*A*) and (*B*). (*G* and *H*) Proliferation curves of the cells studied in (*C*) and (*D*). DOX was withdrawn on day 0. (*I* and *J*) Soft agar colony formation by Kelly cells expressing DOX-inducible wild-type pVHL (*I*) or pVHL L188V (*J*) that were infected to express the indicated sgRNAs and then grown in the presence (+) or absence of DOX (−) for 21 d. For all panels, data presented are means ± SEM; *P value ≤ 0.05, **P value ≤ 0.005, ***P value ≤ 0.005. Two-tailed *P* values were determined by the unpaired *t* test.

We confirmed in low-throughput assays that inactivation of *ARNT*(Fig. 3 *A* and *E*) or *EPAS1*(Fig.3 *B* and *F*), and to lesser extent *HIF1A* (*SI Appendix*, Fig. S5 *A–C*), rescued the proliferation of the exogenous wild-type pVHL cells after DOX withdrawal under standard cell culture conditions. Consistent with the rank order in the primary screen, inactivation of *EPAS1*, and to a lesser extent *ARNT*, also promoted the growth of these cells in soft agar after DOX withdrawal (Fig. 3*I*). *ARNT*, which was the top-scoring enrichment hit in the screen performed with the DOX-inducible pVHL L188V cells, validated in both 2D and 3D growth assays (Fig. 3 *C*, *G*, and *J*). Surprisingly, neither *EPAS1* (Fig. 3 *D*, *H*, and *J*) nor *HIF1A* validated in these secondary assays in the cells deprived of pVHL L188V (*SI Appendix*, Fig. S5 *D–F*). We suspect the former reflects technical differences between the primary screen and secondary assays given the *P* value for *EPAS1* in the primary screen. Finally, inactivating *ARNT* or a *HIFα* family member promoted soft agar growth by the wild-type and L188V pVHL cells even in the presence of DOX, suggesting that suppression of HIF activity by pVHL is incomplete or that elimination of HIF transcriptional activity unmasks a HIF-independent pVHL function that can promote 3D growth (Fig. 3 *I* and *J* and *SI Appendix*, Fig. S5 *C* and *F*).

**Fig. 4.**
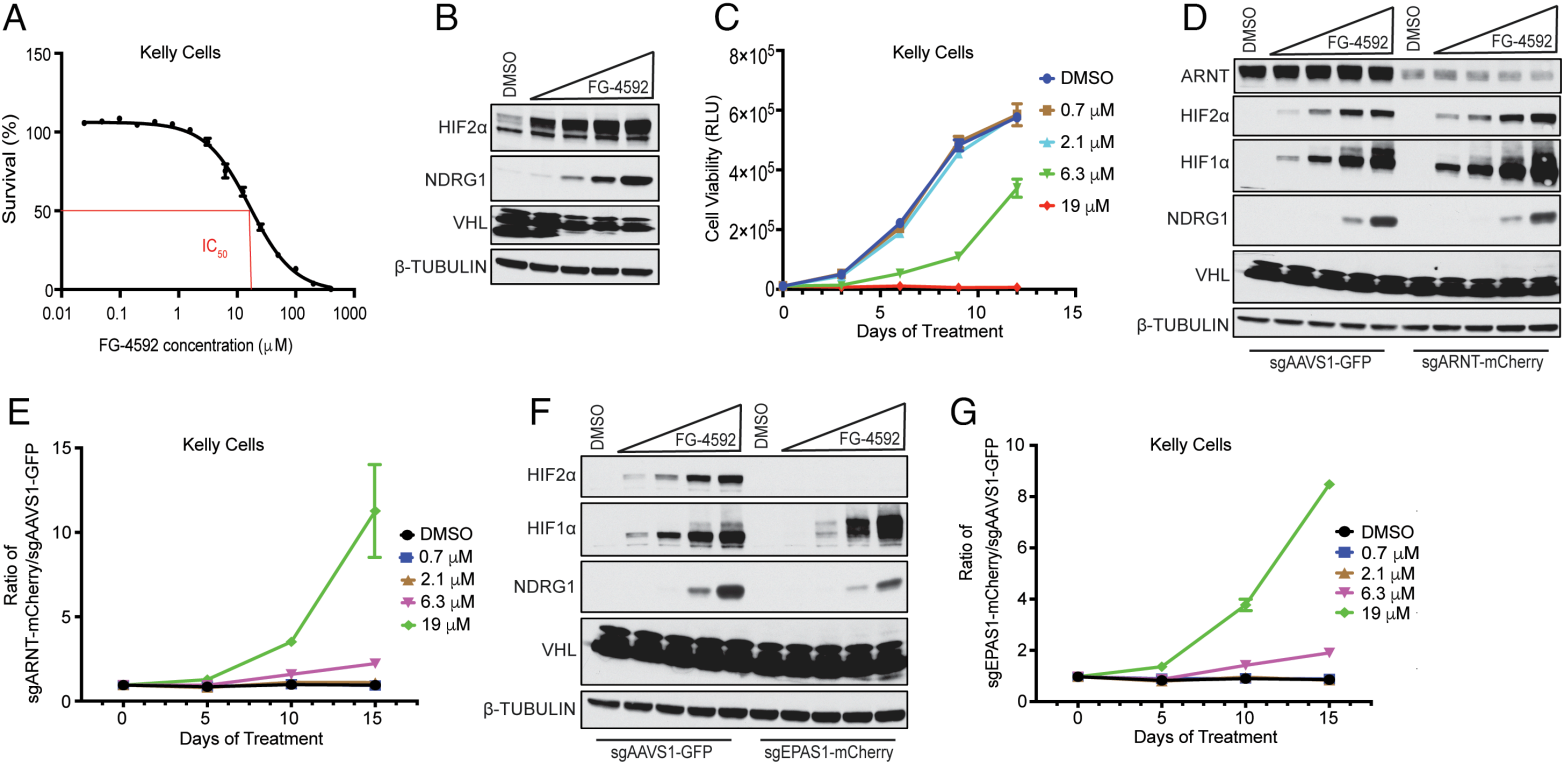
On-target inhibition of Kelly cell proliferation by the HIF stabilizer FG-4592. (*A*) Survival of Kelly cells treated with the indicated concentrations of FG-4592 for 5 d. (*B*) Immunoblots of Kelly cells treated with 0.7 μM, 2.1 μM, 6.3 μM, or 19 μM of FG-4592, as indicated by the triangle, for 6 d. (*C*) Proliferation curves for Kelly cells treated with the indicated concentrations of FG-4592. (*D*–*G*) Competition assays. Kelly cells expressing Cas9 were stably infected to produce a control sgRNA and GFP and either 1) an *ARNT* sgRNA and mCherry (*D* and *E*) or 2) an *EPAS1* sgRNA and mCherry (*F* and *G*). The GFP and mCherry cells were mixed 1:1, treated with the indicated concentrations of FG-4592, and then monitored over time by FACS (*E* and *G*). (*D* and *F*) Immunoblots of the cells in (*E*) and (*G*) after treatment with FG-4592 for 6 d. For all panels, data presented are means ± SEM.

Roxadustat (FG-4592) is an EglN inhibitor that stabilizes both HIF1α and HIF2α (35). It has been approved for the treatment of anemia associated with chronic kidney disease in numerous countries (36–38). FG-4592 at clinically relevant concentrations inhibited the proliferation of Kelly cells (Fig. 4 *A*–*C*). We then infected Cas9-positive Kelly cells to express either 1) mCherry and an *ARNT1* sgRNA or 2) GFP and a control sgRNA (sgAAVS1), mixed them 1:1, treated them with FG-4592, and measured the relative abundance of the two populations by fluorescence-activated cell sorting (FACS) (Fig. 4 *D* and *E*). FG-4592 caused a dose-dependent increase in the percentage of cells with the *ARNT1* sgRNA (Fig. 4 *D* and *E*). In analogous experiments, FG-4592 also promoted the enrichment of Kelly cells expressing an *EPAS1* sgRNA relative to the control cells (Fig. 4 *F* and *G*). Therefore, FG-4592 suppresses the proliferation of Kelly neuroblastoma cells in a HIF2-dependent manner.

Notably, exposure to 1 or 5% oxygen conditions suppressed the proliferation of the parental Kelly cells (*SI Appendix*, Fig. S6*A*) and hypoxia did not rescue the exogenous wild-type pVHL and L188V pVHL expressing Kelly cells after DOX withdrawal (*SI Appendix*, Fig. S6 *B* and *C*). Therefore, the antiproliferative effects of activating HIF2, whether by pVHL loss or FG-4592, were not due solely to the inappropriate activation of HIF2 target genes under normoxic conditions.

**Fig. 5.**
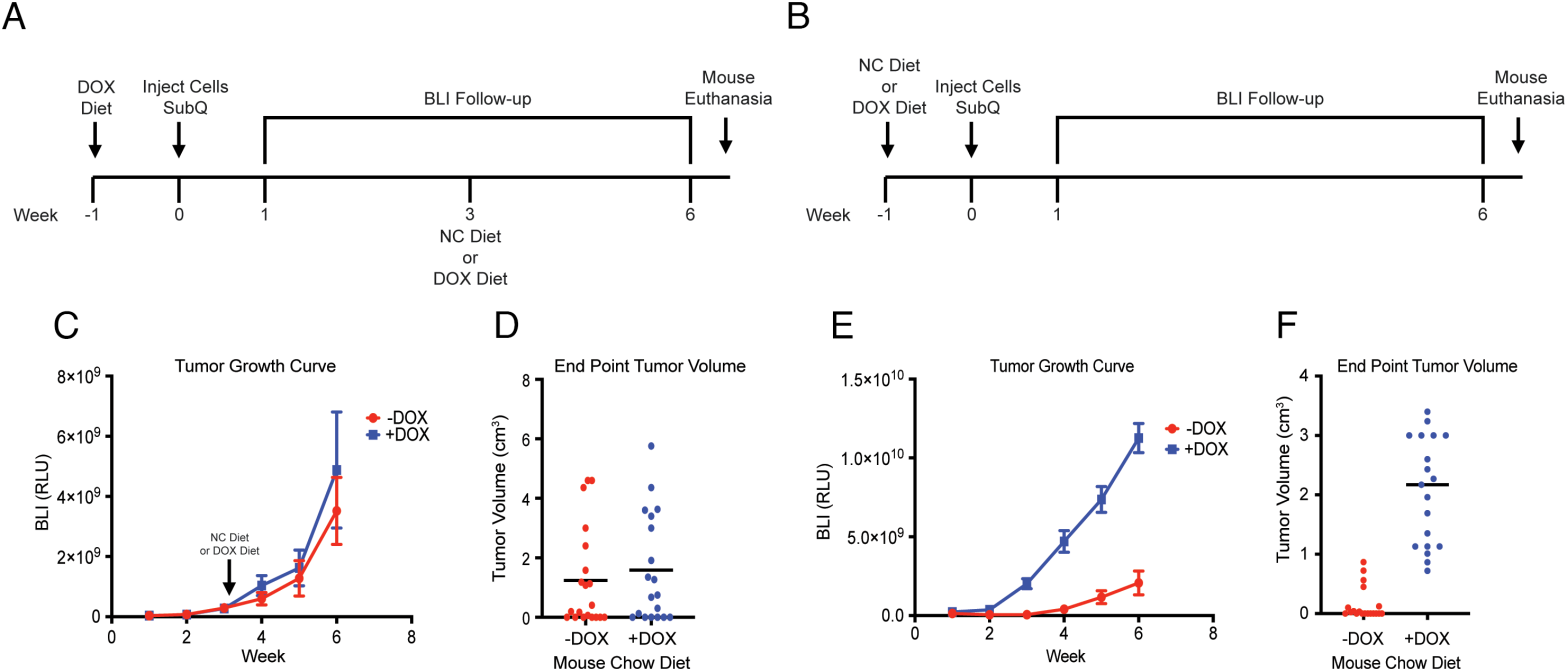
Loss of pVHL attenuates tumor formation by Kelly cells in mice. (*A* and *B*) Schemas: nude mice were injected with Kelly cells expressing firefly luciferase and DOX-inducible wild-type pVHL while on a DOX chow diet. After 3 wk, mice were randomized to chow that either did or did not contain DOX (*A*). In a parallel experiment, nude mice were first placed on chow that either did or did not contain DOX and then subcutaneously injected with Kelly cells expressing firefly luciferase and DOX-inducible wild-type pVHL (*B*). (*C–F*) Tumor growth was measured by serial BLI (*C* and *E*) or by volume measured after excision at necropsies performed at week 7 (*D* and *F*). For all panels, data presented are means ± SEM.

To address the in vivo relevance of our findings, we performed xenograft assays using the Kelly cells described above that lack endogenous pVHL and which express exogenous wild-type pVHL in the presence of DOX. We also introduced a firefly luciferase reporter to enable in vivo bioluminescent imaging (BLI). In the first set of experiments, these cells were injected subcutaneously into nude mice that were maintained on a DOX-containing diet. Once tumors were established, as determined by serial BLI, we randomized the mice to chow that did or did not still contain DOX (Fig. 5*A*). Using this protocol, we did not see an effect of withdrawing DOX on tumor growth (Fig. 5 *C* and *D*). At necropsy, we confirmed upregulation of HIF even in the mice fed DOX (*SI Appendix*, Fig. S7*A*), consistent with the knowledge that macroscopic tumors, especially when grown in the hypovascular subcutaneous space, are profoundly hypoxic.

In the second set of experiments, we randomly injected the Kelly cells into mice that were or were not fed DOX-containing chow (Fig. 5*B*). Using this protocol, we reproducibly saw a marked attenuation of tumor growth in the DOX-free mice (Fig. 5 *E* and *F*). These observations suggest that pVHL loss, and consequent high HIF levels, can suppress the growth of neuroblastoma cells ex vivo and during tumor initiation in vivo. We suspect that the lack of pVHL dependence in established tumors in vivo reflects gradual adaptation over time to changes, such as worsening hypoxia, acidosis, and the influx of inflammatory cells, that occur in tumors as they expand.

The *VHL* gene is ubiquitously expressed and yet *VHL* mutations are linked to a very narrow spectrum of human tumor types. The Broad Institute DepMap data are consistent with the idea that this is because *VHL* inactivation in most cell types causes a loss of cellular fitness, with the cells of origin for VHL-associated neoplasms, such as clear cell renal cell carcinomas, being exceptions.

A notable case is provided by paragangliomas. Individuals with Type 1 germline *VHL* mutations, which are effectively null mutations that cause very high HIF levels, do not develop paragangliomas, in contrast to individuals with Type 2 germline *VHL* mutations, which deregulate HIF to a lesser degree (1). Although there are no faithful models of human paraganglioma, other neural crest–derived tumors, such as neuroblastomas and melanomas, appear to be particularly pVHL-dependent based on the DepMap database. Our data suggest that this fitness defect, at least in the context of Kelly neuroblastoma cells, is due to excessive HIF2α activity. Notably, no single HIF2α target gene scored strongly in our primary screens, strongly suggesting that the growth-suppressive effect of HIF2α in Kelly cells reflects the combinatorial effects of multiple HIF2α target genes and/or a noncanonical HIF2α function. On the other hand, Type 2 *VHL* mutations clearly cause neural crest tumors such as paragangliomas and pancreatic neuroendocrine tumors (26, 39). Moreover, genetic and pharmacological data support that these tumors are driven by HIF2α. These observations, collectively, support a “goldilocks” model wherein increased HIF2α causes paragangliomas and pancreatic neuroendocrine tumors, but HIF2α above a certain threshold prevents the formation of such tumors. It also remains possible that a HIF-independent function of pVHL that is altered by Type 2 *VHL* mutations contributes to paraganglioma formation in conjunction with, and perhaps in some cases instead of, deregulation of HIF2α. Further studies will be required to determine how, mechanistically, high HIF2α levels suppress the proliferation of Kelly cells and whether HIF2α is responsible for pVHL dependence in other cellular contexts. It will also be important to determine whether the most effective way to treat patients with paragangliomas is by lowering HIF2 activity with drugs such as Belzutifan or, paradoxically, by augmenting HIF2 activity with HIF2 agonists.

## Materials and Methods

### Cell Lines.

Kelly cells were obtained from MilliporeSigma and maintained according to the manufacturer's instructions in RPMI 1640 (GIBCO; 11875093), 2 mM Glutamine (GIBCO; 25030081), 10% Tet-Free Fetal Bovine Serum (FBS) (Omega Scientific; FB-15), 100 U/mL Penicillin, and 100 μg/mL Streptomycin (P/S) (Gibco; 25200056). SK-N-FI and SK-N-AS cells were obtained from ATCC and cultured and maintained in DMEM, 0.1 mM Non-Essential Amino Acids (NEAA; GIBCO; 1140050), and 1% P/S. 293T cells were obtained from the Kaelin Laboratory stocks and were maintained in DMEM (GIBCO; 11965092) supplemented with 10% FBS and P/S as above. All cells were maintained in a humidified incubator at 37 °C in an atmosphere containing 5% CO_2_. Kelly, SK-N-FI, and SK-N-AS cells were selected with 600 μg/mL of G418, 200 μg/mL Hygromycin, 10 μg/mL Blasticidin, or 2 μg/mL Puromycin. All cell lines were confirmed to be mycoplasma-free using a MycoAlert Mycoplasma Detection kit (Lonza; LT07-318) and were authenticated by the Promega GenePrint 10 kit for STR profiling (Promega; B9510) in the Molecular Diagnostics Laboratory at the DFCI.

### Plasmids.

A *VHL* cDNA resistant to *VHL* sgRNA#1 was created by site-directed mutagenesis using an Agilent QuikChange II XL Site-Directed Mutagenesis Kit (Agilent; 200522) according to the manufacturer’s instructions on a pDONR223 plasmid containing a wild-type *VHL* cDNA (40). The resulting sgRNA-resistant *VHL* cDNA was then used as the template for further site-directed mutagenesis to create the sgRNA-resistant *VHL* (L188V*), VHL* (C162F), *VHL* (Y112H), or *VHL* (V84L) cDNA. The primers used for site-directed mutagenesis and subcloning are listed below. The pTRIPZ-Gateway-P2A-EGFP-neo vector was made using an In-Fusion Kit (TakaRa; 102518) according to the manufacturer’s instructions by linearizing the pTRIPZ-Gateway-neo (41) with the primers described below in the cloning primers list and then inserting the P2A-EGFP fragment (synthesized by Genewiz). The *VHL* cDNAs from the respective pDONR223 plasmids were then shuttled into pTRIPZ-Gateway-neo vector or pTRIPZ-Gateway-P2A-GFP-neo vector by using Gateway LR Clonase II Enzyme mix (Invitrogen; 11791100) according to the manufacturer’s instructions. The lenti-SpCas9-Hygro vector (Addgene #104995), psPAX2 (Addgene #12260), and pMD2.G (Addgene #12259) were obtained from Addgene. The Lenti-Fluc-Zeo vector was made by shuttling a Fluc cDNA from pDONR223 plasmid into pLX304-Gateway-Zeo (Addgene #160092) using Gateway LR Clonase II Enzyme mix. The Fluc cDNA was amplified from the pLL3.7_EF1a_Fluc-neo vector (Kaelin Laboratory stocks) using the primers described below in the cloning primers list and then shuttled into the pDONR223 vector using Gateway BP Clonase II Enzyme mix (Invitrogen; 11789020) according to the manufacturer’s instructions. The plentiCRISPRv2-Blast vector (Addgene #83480) and plentiGuide-Puro vector (Addgene #52963) were obtained from Addgene. The plentiGuide-GFP-Puro vector and lentiGuide-mCherry-Puro vector (42) were from the Kaelin Laboratory stock. The sgRNA expression vectors were digested with *Bsm*BI (Thermo; ER0451) and ligated to annealed oligonucleotides encoding the desired sgRNAs using T4 ligase (NEB; M0202M) as described in ref. 43. All *VHL* cDNA and sgRNA inserts were validated by DNA Sanger sequencing.

### Targeted Mutagenesis Primers.

*VHL* sgVHL Resistant Forward 5′-cgaagttgagccatacgggtagaactactctcggactgcgattgcagaag-3′

*VHL* sgVHL Resistant Reverse 5′-cttctgcaatcgcagtccgagagtagttctacccgtatggctcaacttcg-3′
  *VHL* L188V Forward 5′-gtggtcttccacatcttcgtagagcgacctgac-3′
  *VHL* L188V Reverse 5′-gtcaggtcgctctacgaagatgtggaagaccac-3′
  *VHL* V84L Forward 5′-tacgggtagaagtactctcggactgcgattgc-3′
  *VHL* V84L Reverse 5′-gcaatcgcagtccgagagtacttctacccgta-3′
  *VHL* Y112H Forward 5′-aggtgacctcggtggctgtggatgcgg-3′
  *VHL* Y112H Reverse 5′-ccgcatccacagccaccgaggtcacct-3′
  *VHL* C162F Forward 5′-cggacaacctggaggaatcgctctttcagagta-3′
  *VHL* C162F Reverse 5′-tactctgaaagagcgattcctccaggttgtccg-3′

### Cloning Primers.

pTRIPZ-GW-neo linearization for In-Fusion cloning Forward 5′-acgcgtggcctccgc-3′

pTRIPZ-GW-neo linearization for In-Fusion cloning Reverse 5′-cttgtcatcgtcatccttgtaatcgatgt-3′

pDONR223- Fluc attB Insert Forward 5′- ggggacaagtttgtacaaaaaagcaggcttaatggaagacgccaaaaacataaagaaag -3′
  pDONR223- Fluc attB insert reverse 5′- ggggaccactttgtacaagaaagctgggttctaca cggcgatctttccgccc-3′

### sgRNA Sequences (including BsmBI Site).

sg*VHL*#1 sense 5′-caccgcatac gggcag cacgacgcg-3′
  sg*VHL*#1 antisense 5′-aaaccgcgtcgtgctgcccgtatg-3′
  sg*ARNT*#3 sense 5′-caccgtggattgtgttggagagtgt-3′
  sg*ARNT*#3 antisense 5′- aaacacactctccaacacaatcca-3′
  sg*ARNT*#4 sense 5′-caccgtggggaacctcacttcgtgg-3′
  sg*ARNT*#4 antisense 5′-aaacccacgaagtgaggttcccca-3′
  sg*EPAS1*#3 sense 5′-caccgactggcaccctatatcccca-3′
  sg*EPAS1*#3 antisense 5′-aaactggggatatagggtgccagt-3′
  sg*EPAS1*#4 sense 5′-caccgtgttctcggagtctagcgca-3′
  sg*EPAS1*#4 antisense 5′-aaactgcgctagactccgagaaca-3′
  sg*HIF1a*#3 sense 5′-caccgaagtgtaccctaactagccg-3′
  sg*HIF1a*#3 antisense 5′-aaaccggctagttagggtacactt-3′
  sg*HIF1a*#4 sense 5′- caccgtatgtgtgaattacgttgtg-3′
  sg*HIF1a*#4 antisense 5′-aaaccacaacgtaattcacacata-3′

### Chemicals.

Doxycycline (Takara; 631311) was reconstituted in sterile water to achieve a stock concentration of 1 mg/mL and added to media to achieve a final concentration of 1 μg/mL. FG-4592 (Selleckchem; S1007) was dissolved in DMSO to achieve a concentration of stock concentration 100 mg/mL and added to media to achieve the desired concentrations.

### Immunoblot Analysis.

Cells were lysed in a buffer containing 50 mM Tris HCl (pH 7.4), 150 mM NaCl, 1 mM EDTA, and 1% Triton X-100 that was supplemented with protease inhibitors (Roche; 11836170001). Protein concentration was quantified using the Bradford Assay (Bio-Rad; 5000006) according to the manufacturer’s instructions. Samples were then mixed with Laemmli SDS-Sample Buffer (6X, Reducing) (Boston BioProducts; BP-11R) to final concentration of 1X and boiled for 10 min prior to loading 20 μg protein per lane onto 10, 12, or 15-well Novex Tris-Glycine Mini 4 to 12% Gradient Gels, WedgeWell format (Invitrogen; 10 wells: XP04120BOX, 12 wells: XP04122BOX, 15 wells: XP04125BOX). Proteins were transferred onto nitrocellulose membranes using the Trans-Blot Turbo Transfer System (Bio-Rad; 1704150) according to the manufacturer's instructions. The membranes were then blocked with 1× TBST (Tween Tris-Buffered Saline [20 mM Tris, 150 mM sodium chloride] with 0.1% Tween-20) supplemented with 5% weight/volume (w/v) powdered nonfat milk for 1 h at room temperature and then incubated overnight at 4 °C with the primary antibody diluted in TBST containing 5% (w/v) Bovine Serum Albumin (GoldBio; A-420-500). The membranes were then washed three times with 1× TBST and then incubated with the appropriate secondary antibody (Jackson ImmunoResearch Lab; Anti-Rabbit: 111-035-003, Anti-Mouse: 115-035-003) diluted in TBST containing 5% (w/v) powdered nonfat milk for 30 min. Following three more washes with 1× TBST, the signal was developed using either Pierce ECL (Thermo Scientific; 32106) or SuperSignal West Pico PLUS (Thermo Scientific; 34580) according to the manufacturer’s instructions. Bound antibodies were visualized with films or digitally using the Azure Biosystems c600 development machine or Bio Rad Chemi Doc MP Imaging System. The primary antibodies used included: Rabbit anti-HIF2α (D6T8V) (Cell Signaling Technology; 29973S), Rabbit anti-NDRG1 (Cell Signaling Technology; 5196S), Rabbit anti-VHL (Cell Signaling Technology; 68547S), Rabbit anti-HA-Tag (C29F4) (Cell Signaling Technology; 3724S), Mouse anti-Cas9 (7A9-3A3) (Cell Signaling Technology; 14697S), Rabbit anti-beta-TUBULIN (Cell Signaling Technology; 2146S), Mouse anti-beta-ACTIN (Cell Signaling Technology; 3700S), Rabbit anti-HIF1α (Cell Signaling Technology; 36169S), Rabbit anti-ARNT (Cell Signaling Technology; 5537S), Mouse anti-Flag-M2 (Sigma Aldrich; F1804-50UG), Rabbit anti-DBH (Cell Signaling Technology; 8586S), Mouse anti-TH (Immunostar Inc.; 22941), and Rabbit anti-HSP90 (Cell Signaling Technology; 4877S).

### Lentivirus Preparation.

To prepare lentiviruses, 1.5 × 10^6^ 293T cells were seeded into a 60 mm plate. The next day 1 μg of lentiviral vector DNA was combined with 0.75 μg of psPAX2 and 0.25 μg of pMD2.G. 250 μL of Opti-MEM media (GIBCO; 31985070) was added to the DNA followed by 6 μL of TransIT-VirusGEN reagent (Mirus; MIR 6700). The mixture was then gently pipetted five times using a p1000 pipetman and subsequently incubated at room temperature for 15 min. Meanwhile, the 293T cell medium was replaced with 3 mL of P/S-free DMEM containing 10% FBS media. The DNA mixture was then added dropwise to the 293T cell media. The cells were then returned to the incubator overnight, followed by media replacement with 4 mL of DMEM containing 30% FBS and 1% P/S. Subsequently, the media containing viruses was collected at 24 h and replaced with fresh DMEM containing 30% FBS and 1% P/S. The media was collected 24 h later and pooled with the early conditioned media. The pooled media was then centrifuged at 755×*g* for 3 min at 25 °C, and then filtered using a 0.45 μm Sterile Syringe Filter (Fisher Scientific; 09-720-517). 1.5 mL aliquots of filtrate were subsequently stored at −80 °C until needed.

### Lentiviral Infection.

For generation of stable cell lines, 2 × 10^6^ cells/well were transferred into 6-well plates with 1.5 mL media per well. 1 mL of lentivirus and 10 μg/mL polybrene (Santacruz; sc-134220) were added per well followed by gentle shaking. The cells were spun at 3,000×*g* for 30 min at 30 °C and then incubated overnight. On the following day, the cells were trypsinized, and transferred to 10 cm plates (1 plate per well) and grown in media supplemented with the drug corresponding to the drug resistance marker for that virus. The conditions for lentiviral sgRNA library screening are described below under *CRISPR-KO Whole Genome Screen*.

### Cell Proliferation Assay.

On day 0, a total of 2,000 cells per well were seeded in black 96-well plates (Costar; 3916) using media containing 1 μg/mL DOX. On day 1, the media were switched to media that did or did not contain DOX. Subsequently, the media were refreshed every 3 d, with either addition or removal of DOX as appropriate. Viable cells were quantified at each time point utilizing the CellTiter-Glo 2.0 Cell Viability Assay (Promega; G9242), following the manufacturer's protocol. At every time point, each experimental condition was represented by 6 wells.

### Cell Cycle Analysis and FITC Annexin V assay.

1 × 10^6^ cells of parental Kelly cells or Kelly cells expressing wild-type pVHL or pVHL L188V were cultured in two 10 cm plates. The following day, the media in each plate was replaced with either DOX-free media or media containing 1 μg/mL DOX. After 3 d, all plates were synchronized by replacing the current media with new media containing 0.1% tet-free FBS, while maintaining the cells in either the absence or presence of DOX. The next day, the media in all plates were changed to 10% tet-free FBS-containing media, keeping the cells in either the absence or presence of DOX. The day after, the cells were washed with 1X PBS and trypsinized. The cells from each plate were divided into two halves. The first half was used for cell cycle analysis using the Cell Cycle Analysis Kit (Sigma-Aldrich; MAK344), following the manufacturer's protocol. The second half of the cells was used to measure apoptosis using the FITC Annexin V Assay Kit (BD Pharmingen; 556420), following the manufacturer's protocol. Color quantification was performed using the SRFortessa cell analyzer (BD Biosciences; 649225) with BD FACSDiva software, and the data were analyzed using FlowJo software.

### Calculating Multiplicity of Infection (MOI) for CRISPR Brunello KO Lentivirus Library.

Kelly wild-type *VHL* or *VHL* L188V cells were trypsinized and counted. 3 × 10^6^ cells in a 2 mL volume were distributed per well in 6-well plates. 10 μg/mL polybrene was added to each well. Subsequently, 0, 10, 20, 30, 40, or 50 μL of CRISPR-ko Brunello lentivirus library, sourced from the Broad Institute (Broad; CP0041) (30), was added to each well. The cells were then centrifuged at 3,000×*g* for 30 min at 30 °C and subsequently incubated overnight at 37 °C. The media for each well was changed the next day. On the following day, each well was trypsinized and transferred to two 10 cm tissue culture plates: one supplemented with 1 μg/mL Puromycin and one without. The cells were maintained for 5 d. Later, cells from each 10 cm plate were trypsinized and counted. To calculate the MOI, the number of cells in the plate supplemented with Puromycin was divided by the number of cells in the plate without Puromycin for each virus volume. The MOI for 30 μL of CRISPR-ko Brunello lentivirus library was found to be 0.3. Throughout the MOI determination, the cells were maintained in media supplemented with 1 μg/mL of DOX.

### CRISPR-KO Whole Genome Screen.

Kelly cells were stably infected with the lentivirus expressing Cas9 and then stably infected with a lentivirus expressing, in a DOX-dependent manner, sgRNA-resistant versions of wild-type *VHL* or *VHL* L188V. These cells were maintained in media supplemented with 1 μg/mL of DOX and then infected with a lentivirus expressing sgVHL#1 to inactivate the endogenous *VHL* gene. A total of 108 × 10^6^ cells were infected per replicate with the CRISPR-ko Brunello lentivirus library, sourced from the Broad Institute (Broad; CP0041) (30), to achieve a coverage of 500 cells/guide. This was done by resuspending the 108 × 10^6^ cells in 72 mL media supplemented with 10 μg/mL polybrene and then adding 1,296 μL of the lentiviral library to achieve a target MOI of 0.3 to 0.4. 3 × 10^6^ cells (2 mL) were then distributed per well in 6-well plates. The cells were then centrifuged at 3,000×*g* for 30 min at 30 °C and subsequently incubated overnight at 37 °C. The media for each well was changed the next day. The following day the cells were trypsinized and transferred to 15 cm tissue culture plates, with each plate being seeded with the combined cells from 6 wells. Puromycin was added to the media at a concentration of 1 μg/mL and the cells were maintained in the puromycin-containing media for 5 d. The surviving cells were grown for another 7 d in the absence of puromycin to allow time for gene editing and for expansion of the edited cells. The cells were then trypsinized and counted. An aliquot of 50 × 10^6^ cells were frozen at −80 °C as the day 14 time point (screen start point). From the remaining cells 50 × 10^6^ cells were cultured in the presence of DOX and 50 × 10^6^ cells were cultured in the absence of DOX. For each arm the cells were trypsinized and counted every 4 d, to maintain 50 × 10^6^ cells in each arm. On day 26 (screen endpoint) all the cells trypsinized, counted, and frozen in 50 × 10^6^ aliquots. The entire screening process was performed in quadruplicate including the treatment arms. Genomic DNA (gDNA) was extracted from the frozen cell pellets using the QIAamp DNA Blood Maxi Kit (50) (Qiagen; 51106) according to the manufacturer's instructions. The concentration of the gDNA was measured using the Qubit dsDNA Quantification Assay Kit (Invitrogen; Q32851) according to the manufacturer’s instructions. The extracted gDNA was then aliquoted into a 96-well plate format according to the Broad Institute instructions that are published on the GPP Web Portal website and sent to the Broad Institute for amplification and sequencing of the enriched sgGuides. The data analysis was conducted using the Pooled Screen Analysis Tool available on the GPP Web Portal (https://portals.broadinstitute.org/gpp).

### Soft Agar Assay.

A stock solution of 3% (w/v) SeaPlaque agarose (Lonza; 50100) in 1× PBS (pH 7.4) was prepared, autoclaved, and stored at room temperature. Immediately prior to its use, the 3% agarose stock solution was melted by brief microwaving and then kept in a 50 °C water bath. To prepare the lower layer, the 3% agarose was diluted to 1% with 37 °C cell culture media and transferred to each well of a 6-well plate (2 mL per well), where it was allowed to solidify at room temperature for 3 h. To prepare the upper layer, the stock solution was diluted to 0.8% with 37 °C cell culture media in a conical tube. The solution was then transferred to another tube that contained an equal volume of 37 °C cell culture media to which had been added 10,000 cells/well. This mixture of cells in 0.4% agarose was gently pipetted and added (1 mL per well) to the 6-well plates containing the lower layers and incubated at room temperature for 3 h. Subsequently, 2 mL of media with or without DOX was added over the solidified agarose wells. In the case of DOX treatment, media were changed every 4 d to maintain consistent DOX concentration throughout the experiment. At the endpoint, colonies were stained with 0.1% Iodonitrotetrazolium chloride diluted in PBS (Sigma; I8377-1G), and the plates were scanned using a photo scanner. Colony quantification was performed using ImageJ.

### IC_50_ Assay.

Cells were seeded at a density of 20,000 cells per well in a black 96-well plate. The following day, media were changed, and FG4592 was added at the desired concentrations. 72 h later, viable cell counts were determined using the CellTiter-Glo 2.0 Cell Viability Assay according to the manufacturer's protocol. Each experimental condition was represented by 6 wells at each time point.

### Color Competition Assay.

Kelly cells expressing Cas9 were infected with either pLenti-sgNegative-GFP, pLenti-sgARNT-mCherry, or pLenti-sgEPAS1-mCherry and then selected using 2 μg/mL puromycin. Subsequently, pLenti-sgNegative-GFP and either pLenti-sgARNT-mCherry or pLenti-sgEPAS1-mCherry cells were combined in a 1:1 ratio. These mixed cells were then cultured in 6-well plates at a density of 1 × 10^5^ cells per well and treated with DMSO or FG4592. On Day 0, 5, 10, and 15, the percentage of GFP-positive or mCherry-positive cells was quantified using the SRFortessa cell analyzer (BD Biosciences; 649225) with BD FACSDiva software.

### Mouse Xenograft Model.

Female NOD.Cg-*Prkdc^scid^ Il2rg^tm1Wjl^*/SzJ (NGS) mice were obtained from The Jackson Laboratory. All animal experimental procedures received approval from the Animal Care and Use Committee of the Dana Farber Cancer Institute. Mice were fed a normal chow diet (-DOX) or, where indicated, a Doxycycline 625PPM 5AW9 Purple diet (TestDiet; 1816024-203). 5 × 10^6^ tumor bioluminescent cells were injected subcutaneously into the one flank per mouse. Tumor growth was assessed weekly using BLI over a 6-wk period. Upon reaching the study endpoint, mice were killed using CO_2_. Subsequently, tumors were collected, and their dimensions were measured using the following formula: Width × Width/(height/2).

## Supplementary Material

Appendix 01 (PDF)

Dataset S01 (XLSX)

Dataset S02 (XLSX)

## Data Availability

All study data are included in the article and/or supporting information. The raw data including the conditions, reference, and PoolQ output files of the genome-wide CRISPR screens for VHL-WT and VHL-L188V Kelly cells are available in this link https://zenodo.org/records/13738229 (31).
