# Supplementary material for: Total loss of VHL gene function impairs neuroendocrine cancer cell fitness due to excessive HIF2α activity
Source: Proc Natl Acad Sci U S A. 2024 Sep 25;121(40):e2410356121. doi: 10.1073/pnas.2410356121 (PMC11459182; doi:10.1073/pnas.2410356121)
Supplement: Supplementary file 1 — Appendix 01 (PDF) [file pnas.2410356121.sapp.pdf]

Supporting Information

Total Loss of *VHL* Gene Function Impairs Neuroendocrine Cancer Cell Fitness Due to Excessive HIF2α Activity

Muhannad Abu-Remaileh[1], Nicole S. Persky[2], Yenarae Lee[2], David E. Root[2], William G. Kaelin, Jr[*1,3]

[1] Department of Medical Oncology, Dana-Farber Cancer Institute, Harvard Medical School, Boston, MA 02215, USA

[2] Broad Institute of MIT and Harvard, 415 Main Street, Cambridge, MA 02142, USA

[3] Howard Hughes Medical Institute, Chevy Chase, MD 20815, USA

* Correspondence should be addressed to William G. Kaelin Jr., William_Kaelin@dfci.harvard.edu

Classification: Biological Sciences; Cell Biology

Figure S1

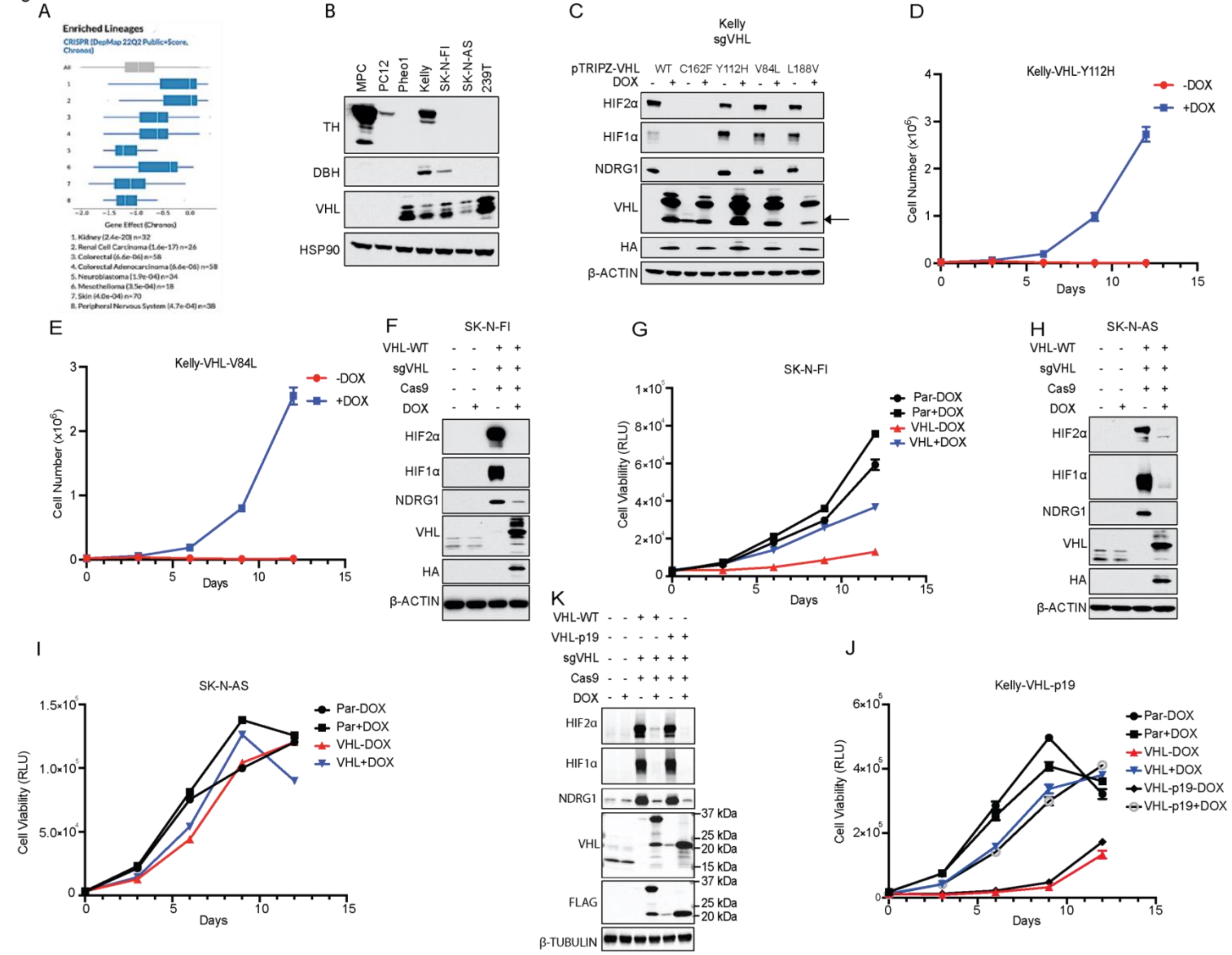

Fig. S1. VHL Dependency and Expression of Neural Crest Markers Across Various Cell Lines. (*A*) Shown are the *VHL* gene effect (chronos) scores, which reflect the dependency on *VHL* based on enrichment or depletion of *VHL* sgRNAs in genome wide CRISPR screens conducted across a large panel of cancer cell lines. A score of 0 indicate a gene is not essential whereas a score of -1 corresponds to the median of all common essential genes. Data acquired by screenshot from www.Depmap.org in April 2021. (*B*) Immunoblot analysis for the indicated cell lines for the neural crest markers Tyrosine Hydroxylase and Dopamine Beta Hydroxylase. (*C*-*E*) Immunoblot analysis (*C*) and proliferation curves (*D* and *E*) of Kelly cells expressing DOX-inducible wild-type pVHL or the indicated pVHL variants. The arrow in (*C*) indicates

endogenous pVHL. (*F* and *G*) Immunoblot analysis (*F*) and proliferation curves (*G*) of SK-N-FI cells. (*H* and *I*) Immunoblot analysis (*H*) and proliferation curves (*I*) of SK-N-AS cells. (*K* and *J*) Immunoblot analysis (*K*) and proliferation curves (*J*) of Kelly cells expressing DOX-inducible wild-type pVHL or pVHL-p19. In (*C*, *F*, *H*, and *K*) the cells were grown in the presence (“+”) or absence of DOX (“-“). In (*D*, *E*, *G*, *I*, and *J*) DOX was or was not withdrawn at time zero. For all panels, data presented are means ± SEM.

Figure S2

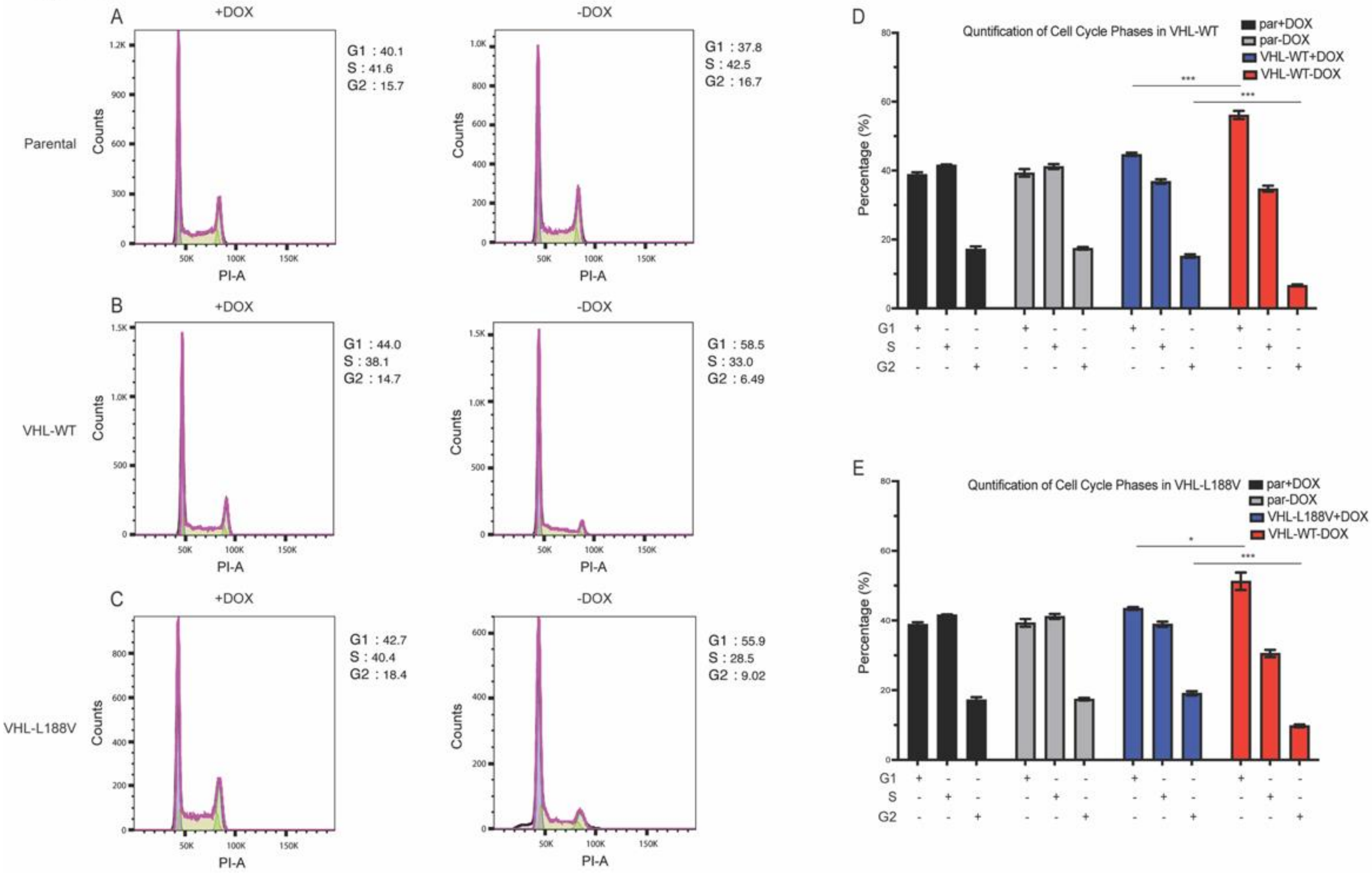

Fig. S2. VHL Inactivation Halts the Cell Cycle of Kelly Neuroblastoma Cells. (*A*-*C*) Representative histograms illustrating the cell cycle distribution of Kelly parental cells and Kelly cells expressing DOX-inducible wild-type pVHL or pVHL L188V, cultured in the presence ("+") or absence of DOX ("-"). The horizontal axis of the histograms represents the PI content, while the vertical axis represents the cell counts. The quantification of each phase is indicated on the right side of each histogram. (*D*-*E*) Bar graph representation of data in (A-C). For all panels, data presented are means ± SEM; * p-value ≤ 0.05, and *** p-value ≤ 0.005. Two-tailed p values were determined by unpaired t-test.

Figure S3

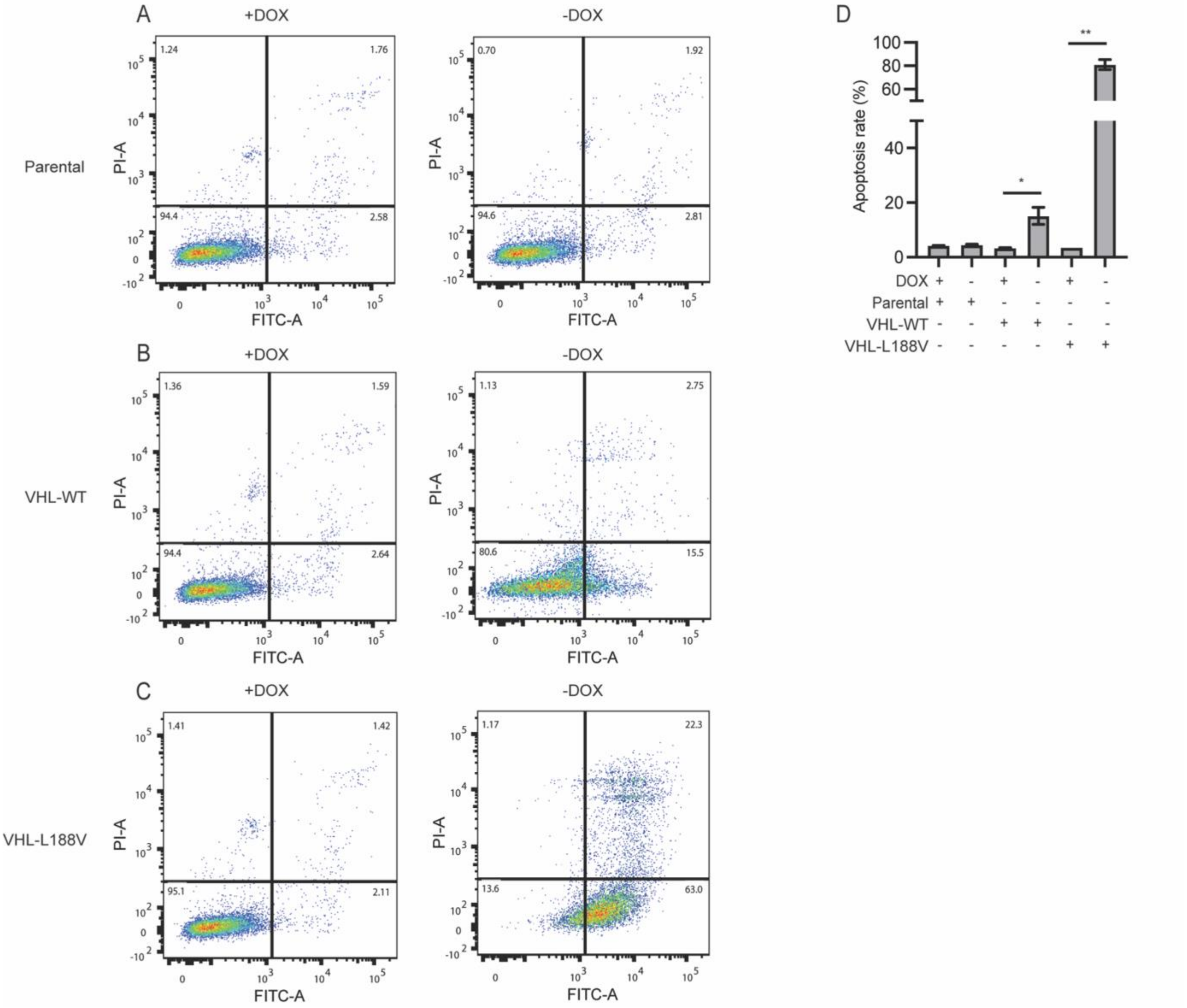

Fig. S3. VHL Inactivation Induces Apoptosis in Kelly Neuroblastoma Cells. (*A*-*C*) Representative cytograms illustrating the distribution of living, proapoptotic, apoptotic, and dead cells in Kelly parental cells and Kelly cells expressing DOX-inducible wild-type pVHL or pVHL L188V. The cells were cultured in the presence ("+") or absence of DOX ("-") and stained with Annexin V/PI. The horizontal axis of the histograms represents Annexin V staining, while the vertical axis represents PI staining. (*D*) Bar graph representation of data (*A*-*C*). For all panels, data presented are means ± SEM; * p value ≤ 0.05, ** p value ≤ 0.005. Two-tailed p values were determined by unpaired t-test.

Figure S4

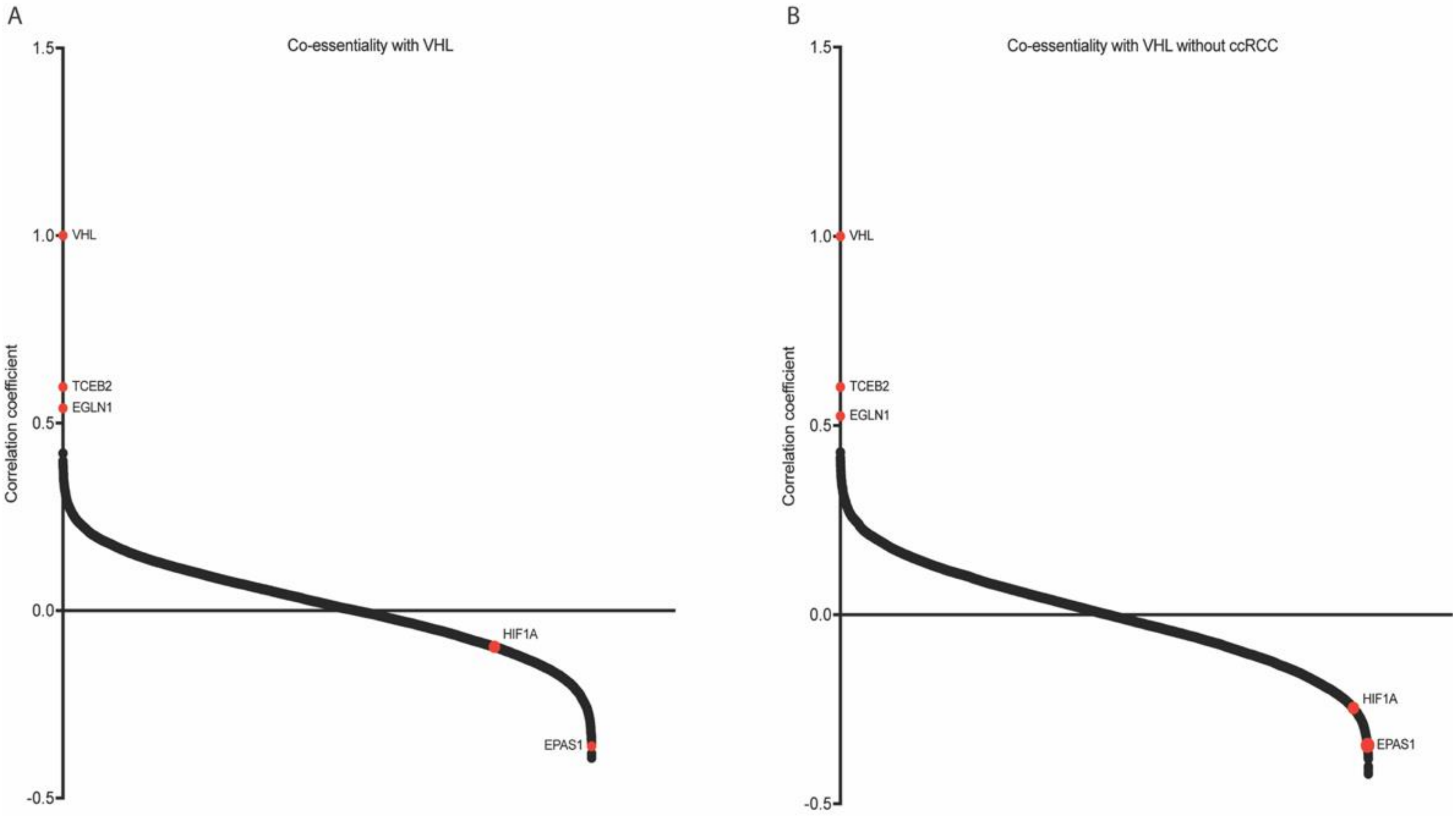

Fig. S4. In Silico Analysis of *VHL* Coessentiality in The Project Achilles Data. (*A*) Coessentiality with *VHL* across all the cells line in the data set. (*B*) Coessentiality with *VHL* across all the cells line in the data set except ccRCCs.

Figure S5

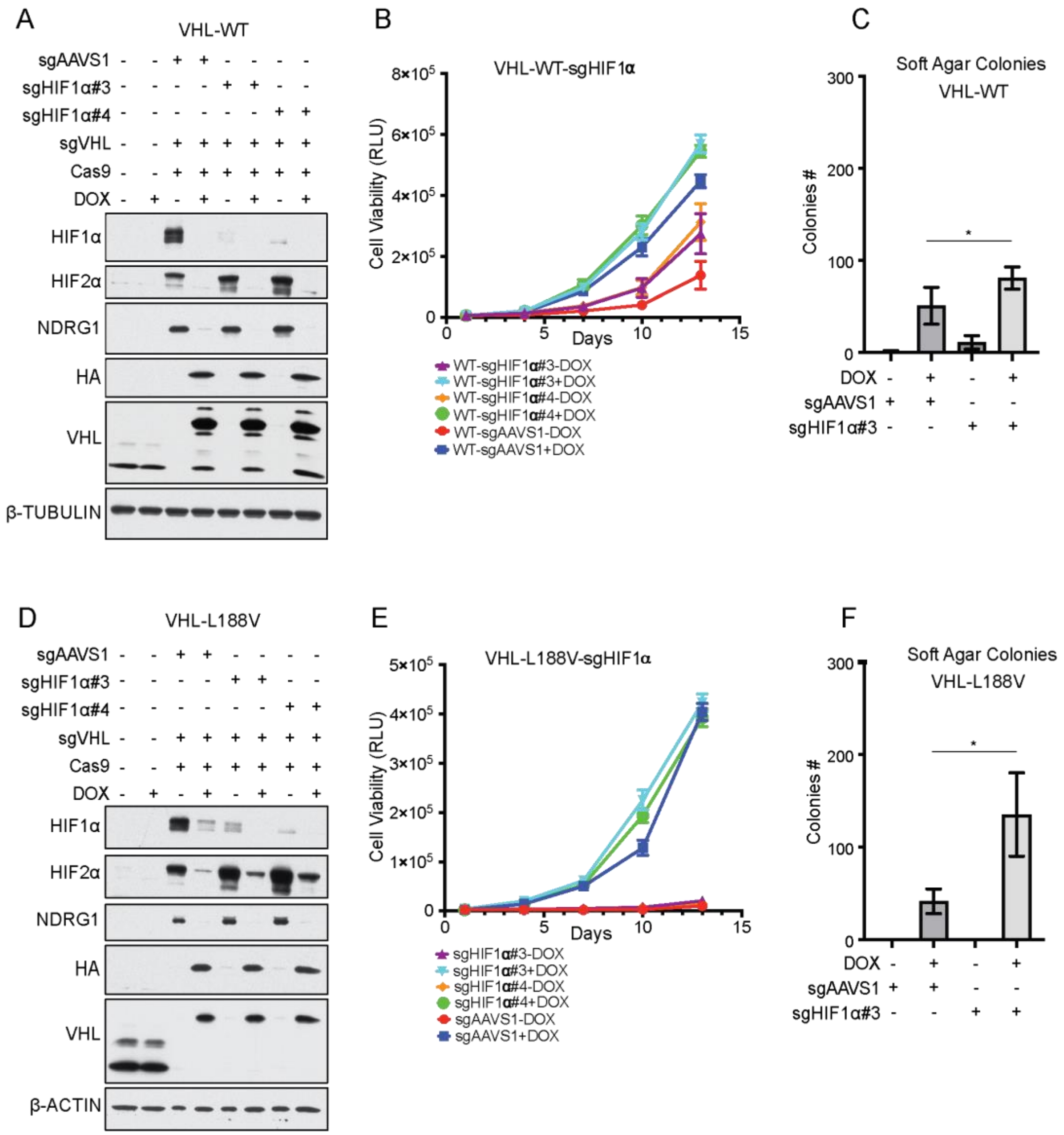

Fig. S5. Partial Rescue of Wild-Type pVHL Loss But not pVHL L188V Loss in Kelly Cells by HIF1α Inactivation. (*A* and *D*) Immunoblot analysis of parental Kelly cells and Kelly cells expressing DOX-inducible wild-type pVHL (*A*) or pVHL L188V (*D*) grown in the presence ("+") or absence of DOX ("-"). Where indicated the cells were also infected to express one of two *HIF1*α sgRNAs or a control *AAVS1* sgRNA. (*B*) Proliferation curves of the cells studied in (*A*). (*E*) Proliferation curves of the cells studied in (*D*). DOX was withdrawn on day zero. (*C* and *F*) Soft agar colony formation by Kelly cells expressing DOX-inducible wild-type pVHL (*C*) or pVHL L188V (*F*) that were infected to express the indicated sgRNAs

and then grown in the presence (“+”) or absence of DOX (“-“) for 21 days. For all panels, data presented are means ± SEM; * p value ≤ 0.05. Two-tailed p values were determined by unpaired t-test.

Figure S6

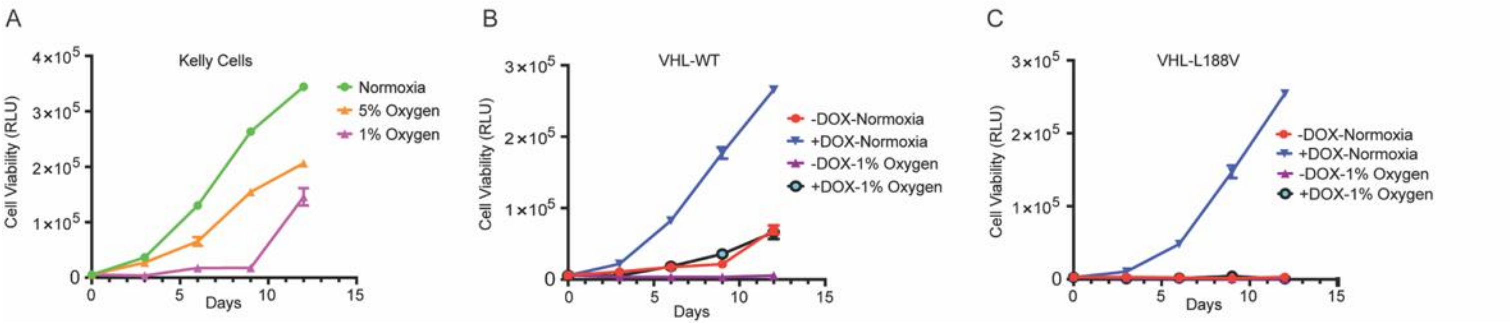

Fig. S6. Hypoxia Suppresses Kelly Cells Proliferation. (*A*) Proliferation curves of parental Kelly Cells incubated under normal, 5% or 1% oxygen levels. (*B* and *C*) Proliferation curves of the Kelly cells expressing DOX-inducible wild-type pVHL (*B*) or pVHL L188V (*C*) grown in the presence ("+") or absence of DOX ("-") and incubated under normal or 1% oxygen levels. DOX was withdrawn on day zero. For all panels, data presented are means ± SEM.

Figure S7

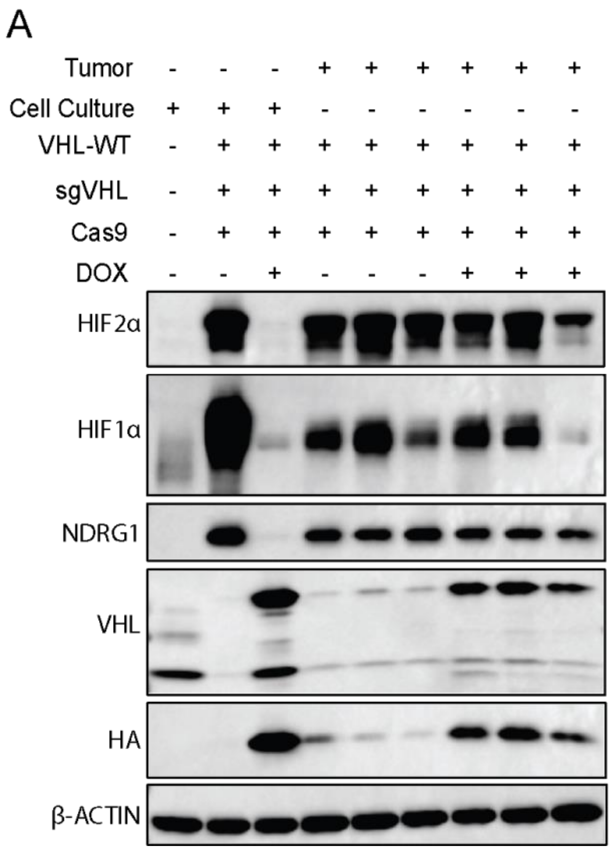

Fig. S7. Upregulation of HIF in Neuroblastoma Xenografts. (*A*) Immunoblot analysis of Kelly cell xenografts formed in mice fed chow that did or did not have DOX as indicated. The Kelly cells were engineered to express exogenous *VHL* in the presence of DOX and lacked endogenous VHL. Parental Kelly cells and Kelly cells expressing DOX-inducible wild-type pVHL, that were grown *in vitro* in the presence (“+”) or absence of DOX (“-“) were used as controls in lanes 1-3.

Table S1. Findings from the genome-wide positive selection screen carried out in wild-type pVHL Kelly cells, associated with Fig 2. Average LFC: Log2 fold changes in guide abundance from the initiation to the termination of the experiment after 12 days of DOX withdrawal. These values are averaged across the four guides linked to each specified gene and four replicates. Average −log (p values): The negative log10 of the p values averaged for each of the four guides related to the specified genes. Perturbations: Sequences of the sgRNAs from the Brunello library corresponding to the specified gene for that row. Individual LFC: Log2 fold change calculated individually for each of the sgRNAs listed in the previous column. Descending ranks: Ranking of each of the four guides based on log2 fold change (enrichment) from the initiation to the termination of the experiment, out of 77,441 total guides in the library.

Table S2. Findings from the genome-wide positive selection screen carried out in L188V pVHL Kelly cells, associated with Fig 2. Average LFC: Log2 fold changes in guide abundance from the initiation to the termination of the experiment after 12 days of DOX withdrawal. These values are averaged across the four guides linked to each specified gene and four replicates. Average −log (p values): The negative log10 of the p values averaged for each of the four guides related to the specified genes. Perturbations: Sequences of the sgRNAs from the Brunello library corresponding to the specified gene for that row. Individual LFC: Log2 fold change calculated individually for each of the sgRNAs listed in the previous column. Descending ranks: Ranking of each of the four guides based on log2 fold change (enrichment) from the initiation to the termination of the experiment, out of 77,441 total guides in the library.
